# The Role of Hydrogen Peroxide and Nitric Oxide in the Induction of Plant-Encoded RNA-Dependent RNA Polymerase 1 in the Basal Defense against *Tobacco Mosaic Virus*

**DOI:** 10.1371/journal.pone.0076090

**Published:** 2013-09-30

**Authors:** Yang-Wen-Ke Liao, Zeng-Hui Sun, Yan-Hong Zhou, Kai Shi, Xin Li, Guan-Qun Zhang, Xiao-Jian Xia, Zhi-Xiang Chen, Jing-Quan Yu

**Affiliations:** 1 Department of Horticulture, Zhejiang University, Hangzhou, People’s Republic of China; 2 Key Laboratory of Horticultural Plants Growth, Development and Quality Improvement, Agricultural Ministry of China, Hangzhou, People’s Republic of China; 3 Department of Botany & Plant Pathology, Purdue University, West Lafayette, Indiana, United States of America; Key Laboratory of Horticultural Plant Biology (MOE), China

## Abstract

Plant RNA-dependent RNA Polymerase 1 (RDR1) is an important element of the RNA silencing pathway in the plant defense against viruses. RDR1 expression can be elicited by viral infection and salicylic acid (SA), but the mechanisms of signaling during this process remains undefined. The involvement of hydrogen peroxide (H_2_O_2_) and nitric oxide (NO) in RDR1 induction in the compatible interactions between *Tobacco mosaic tobamovirus* (TMV) and *Nicotiana tabacum*, *Nicotiana benthamiana*, and *Arabidopsis thaliana* was examined. TMV inoculation onto the lower leaves of *N. tabacum* induced the rapid accumulation of H_2_O_2_ and NO followed by the increased accumulation of *RDR1* transcripts in the non-inoculated upper leaves. Pretreatment with exogenous H_2_O_2_ and NO on upper leaf led to increased *RDR1* expression and systemic TMV resistance. Conversely, dimethylthiourea (an H_2_O_2_ scavenger) and 2-(4-carboxyphenyl)- 4,4,5,5-tetramethylimidazoline-1-oxyl-3-oxide (an NO scavenger) partly blocked TMV- and SA-induced *RDR1* expression and increased TMV susceptibility, whereas pretreatment with exogenous H_2_O_2_ and NO failed to diminish TMV infection in *N. benthamiana* plants with naturally occurring RDR1 loss-of-function. Furthermore, in *N. tabacum* and *A*. *thaliana*, TMV-induced H_2_O_2_ accumulation was NO-dependent, whereas NO generation was not affected by H_2_O_2_. These results suggest that, in response to TMV infection, H_2_O_2_ acts downstream of NO to mediate induction of RDR1, which plays a critical role in strengthening RNA silencing to restrict systemic viral infection.

## Introduction

Most eukaryotes possess an RNA silencing system as a gene regulation and host defense mechanism. This system is activated in cells by double-stranded (ds) RNA, followed by the cleavage of the inducing RNA into short (21- to 24-nucleotide) fragments. These fragments, in turn, mediate multiple regulatory and defense functions in the cells [1], [2]. During this process, RNA-dependent RNA polymerases (RDRs) synthesize dsRNA intermediates, which can be cleaved by different Dicer-like proteins to produce different small interfering RNAs (siRNAs) [3]. Three functionally distinct RDRs have been characterized in tomato, tobacco, and *Arabidopsis* [4]–[6]. RDR1 has been found to be an element of the RNA silencing pathway in the plant defense against viruses and herbivores [6]–[9]. Several recent studies profiled virus-specific small interfering RNAs (vsRNAs) using next-generation sequencing platforms and compellingly implicated plant RDR1 in vsRNA biogenesis and vsRNA-mediated antiviral defense [10]–[12]. RDR1 activity and gene expression in *Arabidopsis* (AtRDR1) and *Nicotiana tabacum* (NtRDR1, the ortholog of AtRDR1) are elicited by viral infection and by salicylic acid (SA) treatment [7], [8]. After infection with a strain of *Potato virus X* (PVX), which does not spread in wild-type tobacco plants, transgenic *NtRDR1* antisense plants accumulated the virus and developed symptoms locally in inoculated lower leaves and systemically in non-inoculated upper leaves [7]. A follow-up study also found that a T-DNA insertion AtRDR1 mutant exhibited markedly enhanced susceptibility to *Tobacco mosaic tobamovirus* (TMV) and *Tobacco rattle virus* [8]. *Nicotiana benthamiana* possesses an *RDR1* gene that contains a 72-nucleotide insert with consecutive in-frame stop codons in the open reading frame and lacks active virus- and SA-inducible function [6]. This natural loss-of-function mutation was named *NbRDR1m* and was suggested to account for the plant’s hypersusceptibility to viruses. However, *N*. *benthamiana* transformed with the SA-inducible *RDR1* gene from *Medicago truncatula* (*MtRDR1*) stilled showed enhanced resistance to tobamoviruses (e.g., TMV, *Turnip vein-clearing virus*, and *Sunn hemp mosaic virus*) but not to viruses outside of the tobamovirus genus (e.g., *Cucumber mosaic virus* (CMV) and PVX) [6]. These results strongly suggest that virus- and SA-inducible RDR1 plays an important role in the plant antiviral defense. However, the details of RDR1 induction and signal transduction during this process remain largely unknown.

In plants, one of the earliest host plant responses to pathogen invasion is an oxidative burst, in which the levels of reactive oxygen species (ROS) increase rapidly [13], [14]. ROS are believed to perform multiple roles during plant defense responses to microbial attack by acting directly in the initial defense and, possibly, serving as central cellular signaling molecules [15]. In particular, hydrogen peroxide (H_2_O_2_) is the most attractive candidate for a ROS signal because of its relatively long half-life and high permeability across membranes [16]. H_2_O_2_ may also play a role in the signaling cascade of virus- and SA-induced RDR1 activation in plants. This idea is supported by several lines of evidence. First, SA application and virus infection resulted in increased H_2_O_2_ concentration, and both are well known RDR1 elicitors [15], [17], [18]. Second, the first identified SA binding protein was a catalase (CAT, EC 1.11.1.6) from tobacco. CAT activity was inhibited by SA in vitro and in vivo, resulting in the generation of H_2_O_2_, which mediated the SA-induced defense response, whereas several antioxidants suppressed the SA-mediated activation of *PR-1* expression and plant defense [15], [19]. Third, a recent study in *Nicotiana glutinosa* plants showed that exogenous H_2_O_2_ induced a significant increase in *NgRDR1* transcripts [13].

In addition to H_2_O_2_, other reactive compounds are also involved in signaling in plants. Nitric oxide (NO) is an emerging essential regulatory molecule in plant immunity and has been well studied. Often, the production of NO and H_2_O_2_ overlaps both spatially and developmentally [20], [21]. Importantly, H_2_O_2_ and NO can react with each other and may influence the activities of enzymes that alter each other’s levels. NO donors caused rapid H_2_O_2_ accumulation in *N*. *tabacum* [22]. In contrast, a NO accumulation mutant *noe1* was also identified that has a defect in a functional rice CAT, resulting in increased H_2_O_2_ in the leaves, which consequently promoted NO production via activation of nitrate reductase [23]. Similarly, in guard cells of *Arabidopsis* plants, NO synthesis and stomata closure in response to abscisic acid (ABA) were severely reduced in the H_2_O_2_-generation-related NADPH oxidase double mutant *atrbohD/F* [24]. Furthermore, a previous study also showed that virus infection and SA activated NO synthesis [25], [26]. Collectively, these studies suggest that H_2_O_2_ and NO generation may be related to virus- and SA-induced *RDR1* induction in plants. However, the potential cross talk between H_2_O_2_ and NO, as well as its function in plant antiviral processes, has not yet been resolved.

In the present study, using pharmacological and biochemical approaches, we investigated the potential role of H_2_O_2_ and NO in virus- and SA-induced RDR1 activation and RDR1 function in the compatible interactions between TMV and the model host plants *N*. *tabacum*, *N*. *benthamiana*, and *Arabidopsis*. We found that *RDR1* expression was elevated by H_2_O_2_ and NO donors, whereas TMV- and SA-induced *RDR1* transcript levels were compromised by H_2_O_2_ and NO scavengers in all three plant species. Employing *Arabidopsis* mutants that were defective in H_2_O_2_ or NO synthesis and transgenic *N*. *benthamiana* plants transformed with SA-inducible *MtRDR1* from *M. truncatula* [6], we showed that H_2_O_2_ may function downstream of NO and may mediate the induction of *RDR1* in response to TMV challenge, thereby limiting the systemic infection and accumulation of the virus. These results provide initial insights into the signaling mechanisms underpinning virus- and SA-induced *RDR1* activation in host plants.

## Results

### Changes of *NtRDR1* expression and H_2_O_2_ and NO accumulation in *N. tabacum* after TMV inoculation

**Figure 1:**
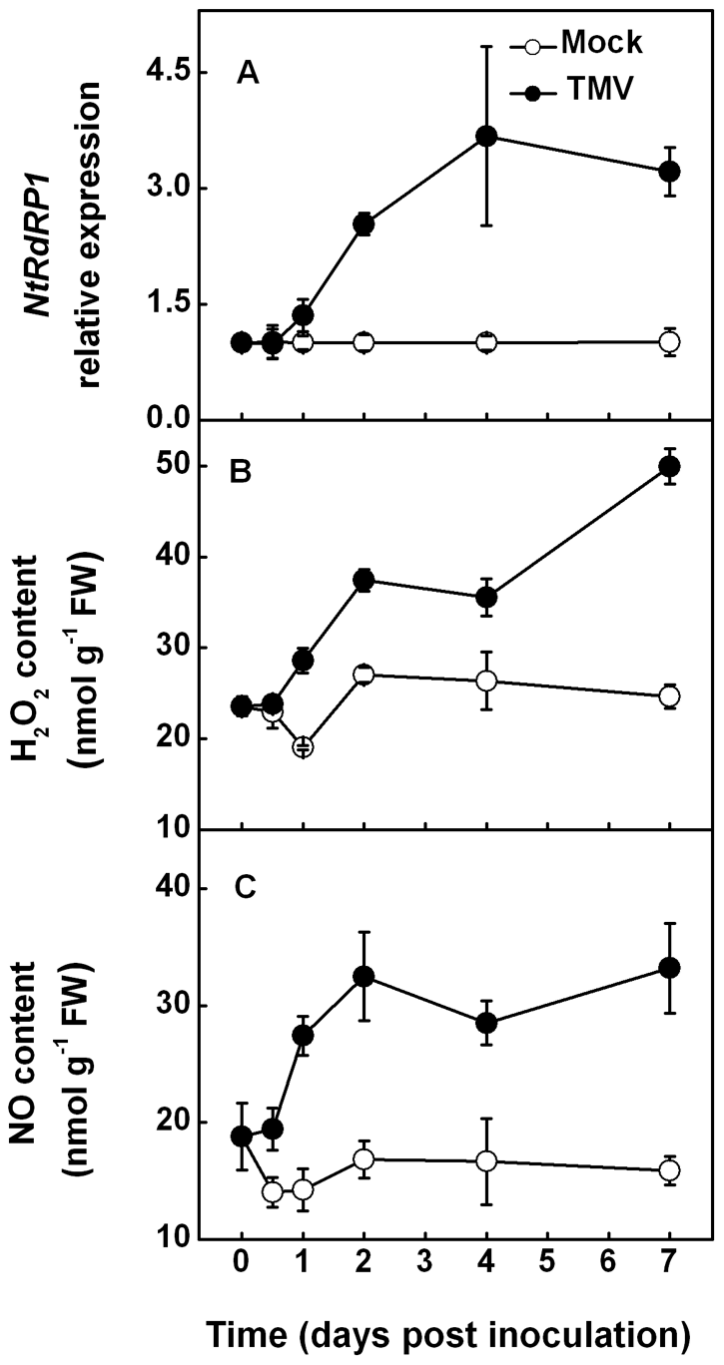
Time course changes of *NtRDR1* transcripts and concentrations of H_2_O_2_ and NO. Changes in the transcript levels of *Nicotiana tabacum RNA-dependent RNA Polymerase 1* (*NtRDR1*, **A**), hydrogen peroxide (H_2_O_2_, **B**), and nitric oxide (NO, **C**) over time in the non-inoculated upper leaves of *Nicotiana tabacum* plants after TMV inoculation of the lower leaves. The results are expressed as the mean ± SD, *n*  =  4.

*NtRDR1* relative expression and the quantity of H_2_O_2_ and NO were determined in the compatible interactions between TMV and *N*. *tabacum* plants (Figure 1). In non-inoculated upper leaves, *NtRDR1* relative expression began to increase 2 days post inoculation (dpi) and reached a maximum induction of 3.7-fold at 4 dpi. Concomitant with the induction of *NtRDR1* expression, we observed a rapid increase in the levels of H_2_O_2_ and NO signaling compounds, which resulted in significant differences over mock-treated plants at 1 and 0.5 dpi, respectively. Furthermore, the increased relative expression of *NtRDR1* and increased concentration of H_2_O_2_ and NO resulting from TMV inoculation remained high until the end of the experiment. At 7 dpi, the relative expression of *NtRDR1* and the concentration of H_2_O_2_ and NO were 2.2-, 1.0-, and 1.1-fold higher than those in mock-inoculated plants, respectively.

### TMV- and SA-induced *RDR1* induction is enhanced by exogenous H_2_O_2_ and NO, and is reversed by scavengers

**Figure 2:**
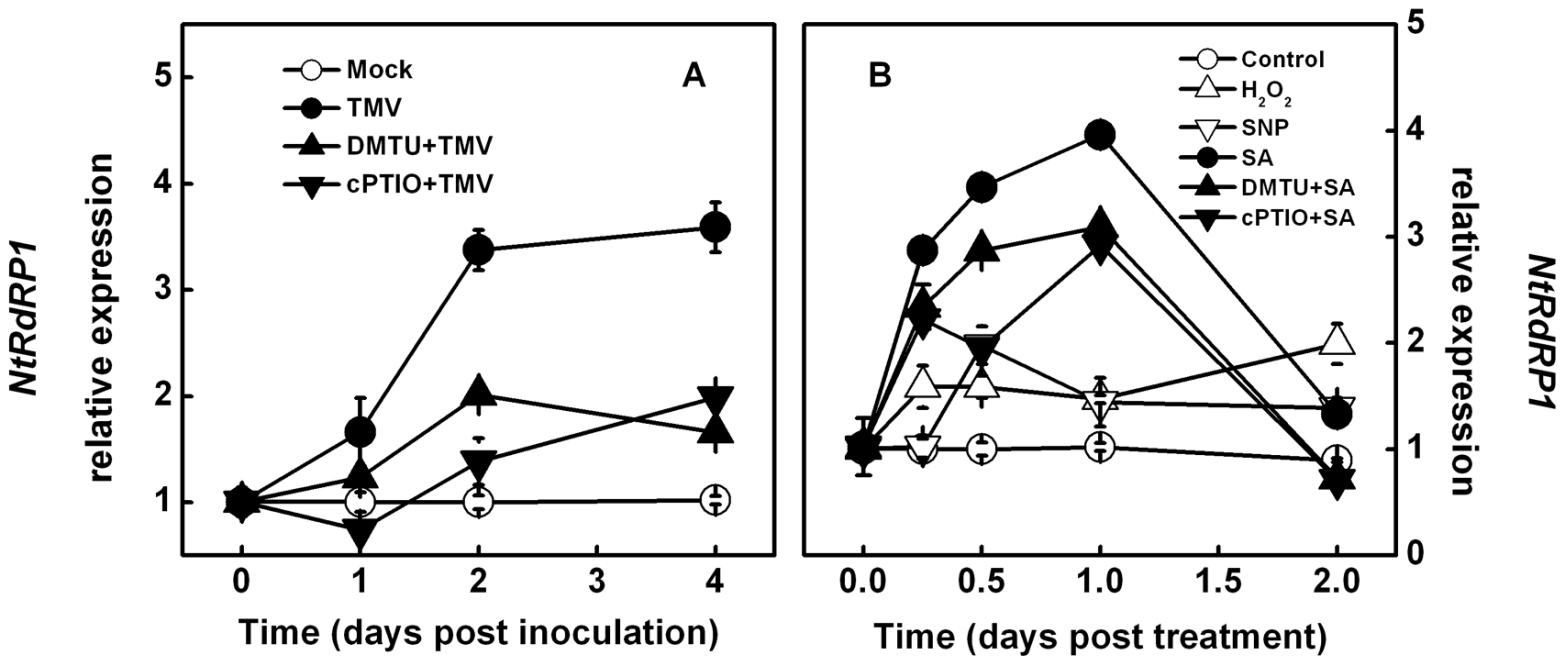
Effects of TMV infection, SA, and different chemical pretreatments on *NtRDR1* transcripts. **A**. Changes in TMV-induced *NtRDR1* transcript levels over time in non-inoculated upper leaves induced by hydrogen peroxide (H_2_O_2_) and nitric oxide (NO) scavengers. The non-inoculated upper leaves were subjected to DMTU (H_2_O_2_ scavenger) or cPTIO (NO scavenger) pretreatment 12 hours before TMV inoculation. **B**. Changes in SA-induced *NtRDR1* transcript levels over time caused by exogenous H_2_O_2_, NO, and their scavengers. The results are expressed as the mean ± SD, *n*  =  4.

To explore the possible involvement of H_2_O_2_ and NO in *RDR1* induction, we pretreated non-inoculated upper leaves of *N*. *tabacum* plants with dimethylthiourea (DMTU, a H_2_O_2_ and OH· scavenger) and 2-(4-carboxyphenyl)-4,4,5,5- tetramethylimidazoline-1-oxyl-3-oxide (cPTIO, a NO scavenger) 12 hours before TMV inoculation. TMV-induced *NtRDR1* expression was compromised by DMTU and cPTIO application, showing a reduction of approximately 50% with both chemical modulators at 4 dpi (Figure 2A). Next, we applied the exogenous H_2_O_2_, the NO chemical donor sodium nitroprusside (SNP), and SA to *N*. *tabacum* leaves, and *NtRDR1* expression was analyzed throughout a 2-day experiment. Exogenous H_2_O_2_ and SNP induced a significant increase in *NtRDR1* transcript levels (Figure 2B). Furthermore, the SA-induced increase in *NtRDR1* transcript levels was substantially suppressed by elimination of H_2_O_2_ and NO.

**Figure 3:**
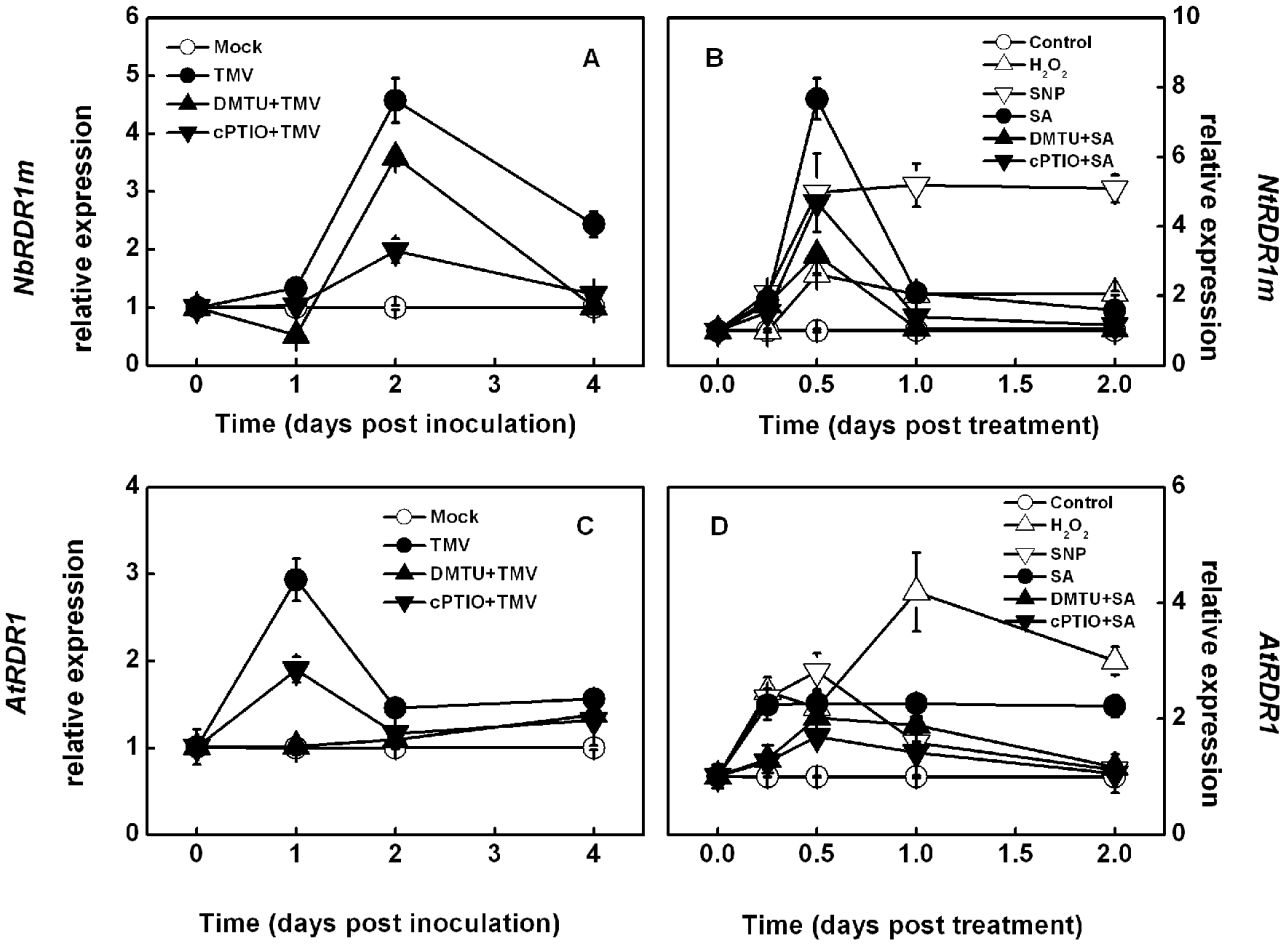
Effects of TMV infection, SA, and different chemical pretreatment on transcripts of *NbRDR1m* and *AtRDR1*. **A** and **C**. Changes in TMV-induced *NbRDR1m* (**A**) and *AtRDR1* (**C**) transcript levels over time in non-inoculated upper leaves induced by scavengers of hydrogen peroxide (H_2_O_2_) and nitric oxide (NO). The non-inoculated upper leaves were subjected to DMTU (H_2_O_2_ scavenger) or cPTIO (NO scavenger) pretreatment 12 hours before TMV inoculation. **B** and **D**. Changes in SA-induced *NbRDR1m* (**B**) and *AtRDR1* (**D**) transcript levels over time induced by exogenous H_2_O_2_, NO, and their scavengers. The results are expressed as the mean ± SD, *n*  =  4.

To determine the involvement of H_2_O_2_ and NO in TMV- and SA-induced *RDR1* in other plants, the same experiments were also performed with *N*. *benthamiana* and *Arabidopsis* plants. Similar to the results obtained with *N*. *tabacum*, DMTU and cPTIO significantly reduced TMV- and SA-induced *NbRDR1m* and *AtRDR1* relative expression (Figure 3), whereas exogenous H_2_O_2_ and SNP application resulted in increased *NbRDR1m* and *AtRDR1* transcript levels.

### TMV-induced H_2_O_2_ synthesis and associated *RDR1* expression are reduced by NO elimination and reversed by exogenous NO application, but H_2_O_2_ does not influence TMV-induced NO evolution

**Figure 4:**
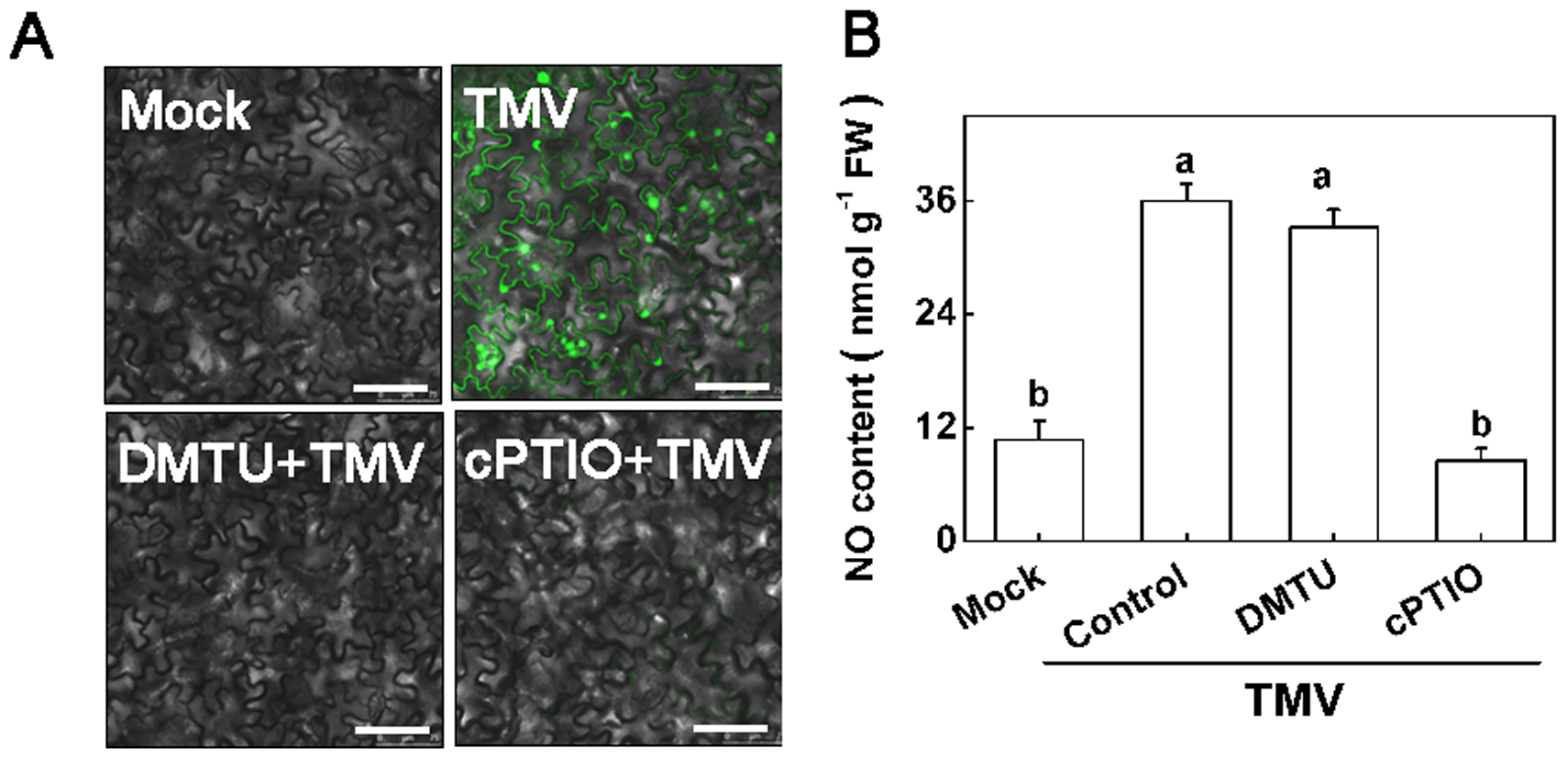
Effects of scavengers of H_2_O_2_ and NO on TMV-induced H_2_O_2_ and NO accumulation in *Nicotiana tabacum* *.* The non-inoculated upper leaves were subjected to DMTU (H_2_O_2_ scavenger) or cPTIO (NO scavenger) pretreatment 12 hours before TMV inoculation. **A**. Representative H_2_O_2_ visualization using H_2_DCF-DA and a confocal microscope in which H_2_O_2_ exhibited green fluorescence. All images are at the same magnification and the white scale bar indicates 75 µm. **B**. NO assayed with Griess reagent. The results are expressed as the mean ± SD, *n* = 4. The letters indicate significant differences between the treatments (*P*<0.05).

To study the relationship between H_2_O_2_ and NO in *RDR1* induction under TMV inoculation conditions, we treated the non-inoculated upper leaves of *N*. *tabacum* plants with DMTU and cPTIO, followed by inoculation with TMV inoculum on the lower leaves. At 2 dpi, a confocal laser scanning microscope (CLSM) was used to detect changes in H_2_O_2_ in the leaves. There was an increase in the green fluorescence H_2_O_2_ signal in TMV-inoculated plants compared to mock-inoculated plants (Figure 4A). The TMV-induced H_2_O_2_ accumulation was greatly suppressed by its scavenger DMTU. Interestingly, the NO scavenger cPTIO also completely inhibited H_2_O_2_ accumulation (Figure 4A). In contrast, NO quantification using Griess reagent showed that NO generation from TMV attack was only http://dict.cnki.net/dict_result.aspx?searchword=%e5%bd%bb%e5%ba%95&tjType=sentence&style=&t=thoroughlyblocked by cPTIO and not by H_2_O_2_ elimination (Figure 4B).

**Figure 5:**
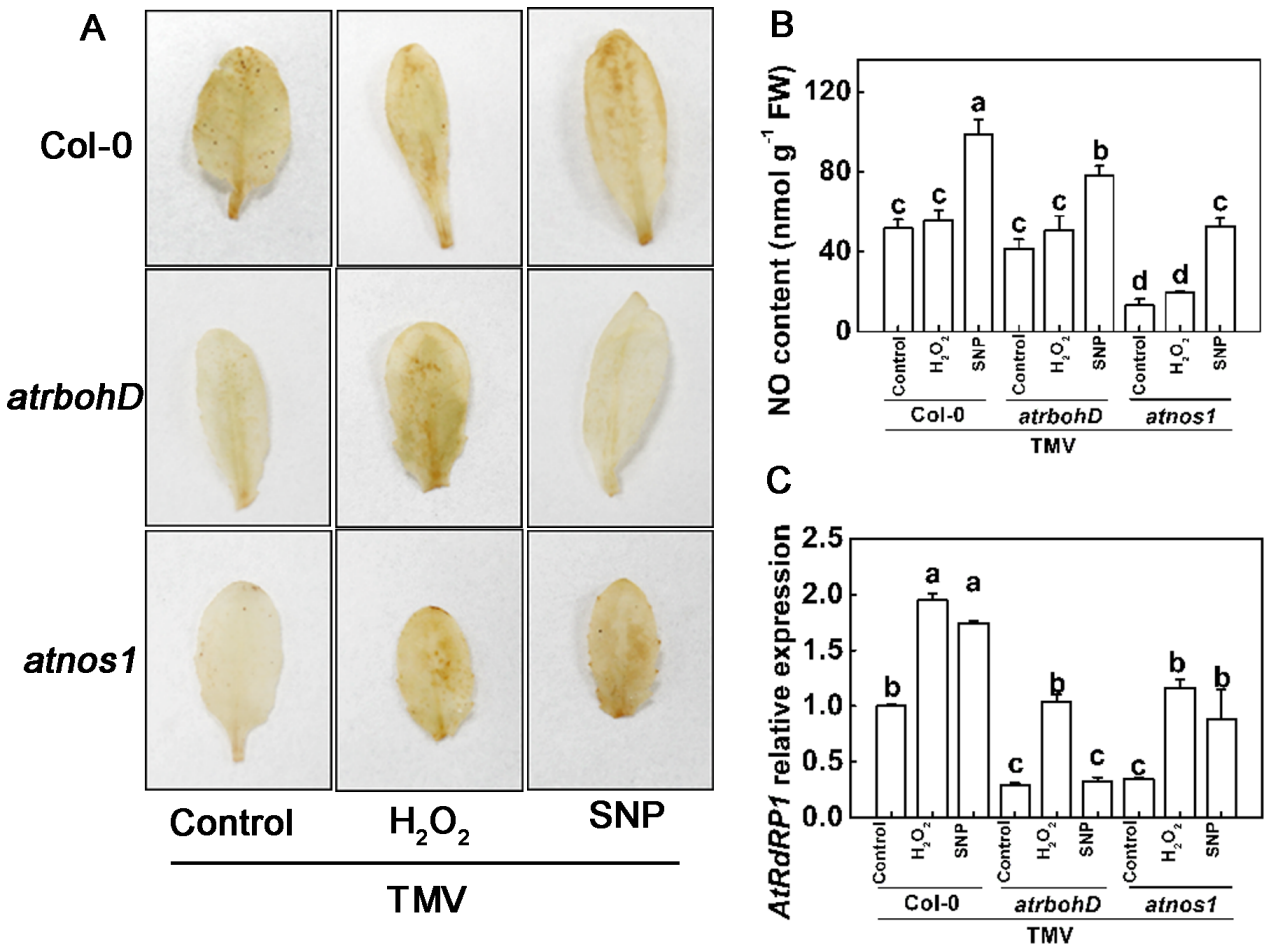
Relationship of H_2_O_2_, NO, and *AtRDR1* transcripts in wild-type and mutant *Arabidopsis* plants. Effects of TMV inoculation and exogenous application of hydrogen peroxide (H_2_O_2_) and nitric oxide (NO) on endogenous H_2_O_2_ and NO concentrations and on transcript levels of *RNA-dependent RNA Polymerase 1* in non-inoculated upper leaves of wild-type and mutant *Arabidopsis* plants (*AtRDR1*). The non-inoculated upper leaves were pretreated with H_2_O_2_ or SNP 12 hours before TMV inoculation, and leaf samples were collected at 2 days post inoculation. **A**. H_2_O_2_ detection using DAB histochemical staining. **B**. NO concentration. **C**. *AtRDR1* transcripts. The results are expressed as the mean ± SD, *n*  =  4. The letters indicate significant differences between the treatments (*P*< 0.05).

To establish a more direct relationship between H_2_O_2_ and NO in TMV-induced *RDR1* expression, we collected the *Arabidopsis* H_2_O_2_ synthetic enzymatic mutant *atrbohD*, the mutant *atnoa1* that is indirectly impaired in NO synthesis, and the wild-type Columbia (Col-0) ecotype. We pretreated the plants with exogenous H_2_O_2_ and SNP on the non-inoculated upper leaves followed by TMV inoculation on the lower leaves. The *in planta* concentration of H_2_O_2_ and NO as well as *RDR1* transcript levels in the non-inoculated upper leaves were analyzed at 2 dpi. In wild type Col-0 plants, pretreatment with either H_2_O_2_ or the NO donor SNP resulted in a significant increase in H_2_O_2_ histochemical staining compared to untreated plants (Figure 5), whereas the NO concentration was elevated by SNP pretreatment but not by exogenous H_2_O_2_. As expected, TMV-induced H_2_O_2_ accumulation was abolished in *atrbohD* http://www.arabidopsis.org/servlets/TairObject?id=132871&type=locusplants and was increased when exogenous H_2_O_2_ was applied. However, this effect was not observed in SNP-pretreated plants. At the same time, the NO concentration in *atrbohD* http://www.arabidopsis.org/servlets/TairObject?id=132871&type=locusplants was comparable to that in Col-0 plants, and no significant increase was observed after exogenous H_2_O_2_ treatment. In contrast, the H_2_O_2_ concentration in *atnoa1* http://www.arabidopsis.org/servlets/TairObject?id=40002&type=locusplants was greatly reduced compared to that in Col-0 plants. However, the H_2_O_2_ concentration increased when either exogenous H_2_O_2_ or SNP was applied. The impaired NO accumulation in *atnoa1* plants recovered to levels observed in the Col-0 control level after SNP application but showed no significant changes in response to exogenous H_2_O_2_ treatment. Furthermore, the changes of *in planta* H_2_O_2_ and NO concentration were closely related to *RDR1* induction. In contrast to the further increase in TMV-induced *AtRDR1* expression by exogenous H_2_O_2_ and SNP in Col-0 plants, *AtRDR1* transcript levels in the leaves of *atrbohD* http://www.arabidopsis.org/servlets/TairObject?id=132871&type=locusand *atnoa1* http://www.arabidopsis.org/servlets/TairObject?id=40002&type=locusplants showed a decrease of approximately 70% compared to the levels in Col-0 leaves. The lower *AtRDR1* transcript levels in these mutants were greatly increased with exogenous H_2_O_2_ treatment, whereas SNP pretreatment only increased *AtRDR1* transcript levels in *atnoa1* http://www.arabidopsis.org/servlets/TairObject?id=40002&type=locusplants and not *atrbohD* http://www.arabidopsis.org/servlets/TairObject?id=132871&type=locus plants.

### Function of H_2_O_2_- and NO-associated *RDR1* induction in the basal antiviral defense

**Figure 6:**
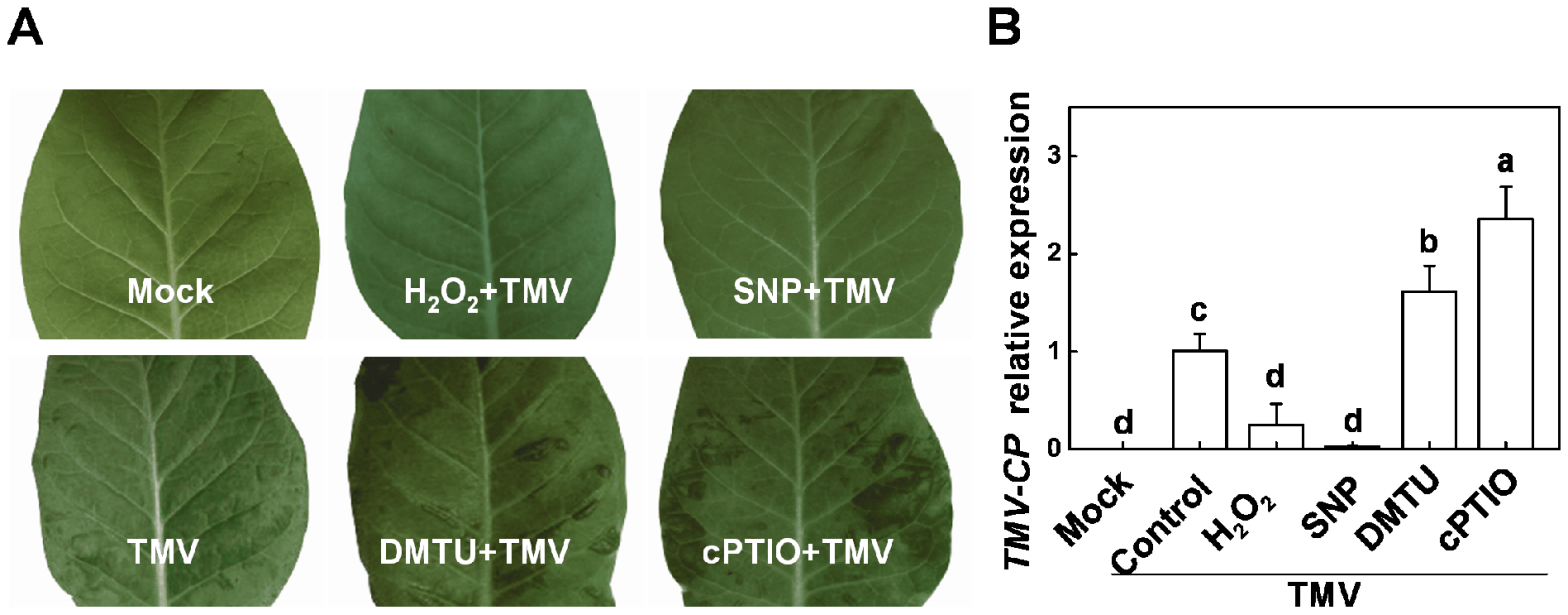
Effects of chemical pretreatments on TMV infection in *Nicotiana tabacum* plants . Effects of different chemical pretreatments and TMV infection on TMV symptom development and *TMV-CP* transcription in the non-inoculated upper leaves of *Nicotiana tabacum* plants. The non-inoculated upper leaves were subjected to application of hydrogen peroxide (H_2_O_2_) or nitric oxide (NO) chemical modulators 12 hours before TMV inoculation. **A**. Symptoms were photographed at 27 days after inoculation. **B**. *TMV-CP* transcript levels were analyzed by real-time reverse-transcription polymerase chain reaction at 7 days after inoculation. The results are expressed as the mean ± SD, *n*  =  4. The letters indicate significant differences between the treatments (*P*< 0.05).

We then tested whether the H_2_O_2_- and NO-associated *RDR1* induction functions in the basal defense against TMV inoculation. Exogenous H_2_O_2_ and NO chemical modulators were applied on the non-inoculated upper leaves of *N*. *tabacum* plants 12 hours prior to TMV inoculation. The symptoms of systemic leaves were then photographed at 27 dpi (Figure 6A). The TMV infection clearly resulted in visible damage with necrotic lesions and crinkling of leaves. Pretreatment with H_2_O_2_ or SNP significantly decreased TMV susceptibility, and these leaves did not show any visible systemic symptoms. In contrast, DMTU or cPTIO pretreatment substantially increased the TMV susceptibility and led to the development of enhanced necrotic lesions and crinkling of leaves. Moreover, the levels of *TMV-CP* mRNA in non-inoculated upper leaves analyzed by RT-PCR at 7 dpi correlated highly with the observed symptoms (Figure 6B). Compared to plants that received the TMV challenge alone, H_2_O_2_ and SNP pretreatment reduced *TMV-CP* mRNA by 75% and 98%, respectively, whereas DMTU and cPTIO pretreatment markedly increased these levels.

**Figure 7:**
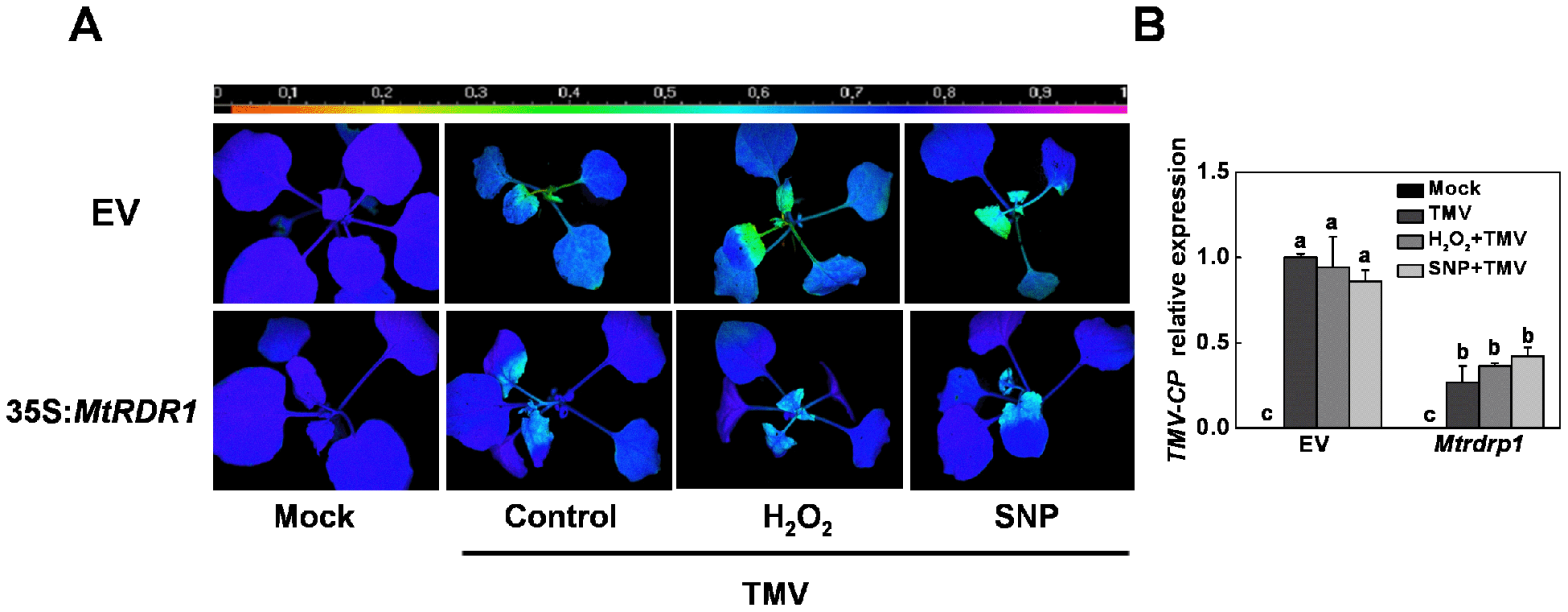
Effects of exogenous H_2_O_2_ and NO on TMV infection in *Nicotiana benthamiana* plants. Effects of exogenous application of hydrogen peroxide (H_2_O_2_) and nitric oxide (NO) and TMV inoculation on the maximum photochemical efficiency of photo system II (Fv/Fm) and *TMV-CP* gene transcription in the non-inoculated upper leaves of empty vector (EV)-transformed and *MtRDR1*-transformed *Nicotiana benthamiana* plants. The non-inoculated upper leaves were pretreated with hydrogen peroxide (H_2_O_2_) or nitric oxide (NO) 12 hours before TMV inoculation. **A**. Chlorophyll fluorescence images of the maximum quantum yield of PSII (Fv/Fm) at 7 days post inoculation. The color gradient scale at the top indicates the magnitude of the fluorescence signal represented by each color. **B**. *TMV-CP* transcript levels analyzed by real-time reverse-transcription polymerase chain reaction at 7 days post inoculation. The results are expressed as the mean ± SD, *n*  =  4. The letters indicate significant differences between the treatments (*P*< 0.05).

To test whether H_2_O_2_- and NO-associated RDR1 has a general function in different species, we also applied exogenous H_2_O_2_ and NO to transgenic *N*. *benthamiana* plants transformed with the SA-inducible *MtRDR1* gene from *M. truncatula* or an empty vector (EV) as a control. A chlorophyll fluorescence imaging method was used to analyze the response of the maximum photochemical efficiency of PSII in the dark-adapted state (Fv/Fm) to the H_2_O_2_ and NO chemical modulators in TMV-infected *N*. *benthamiana* plants. At 7 dpi, TMV inoculation resulted in a significantly lower Fv/Fm in the central leaves of EV control plants relative to their *MtRDR1* transgenic counterpart (Figure 7A), which indicated that TMV damaged EV plants more severely than it damaged *MtRDR1*-transformed plants. Furthermore, there were no evident effects of H_2_O_2_ or NO on TMV susceptibility in EV and transgenic plants. *TMV-CP* transcripts assayed at the same time showed the same pattern as the chlorophyll fluorescence imaging (Figure 7B), which showed that without the significant effects of H_2_O_2_ or SNP, the *TMV-CP* mRNA level in non-inoculated upper leaves of EV plants was approximately 2-fold higher than that in leaves of *MtRDR1* transformed plant.

**Figure 8:**
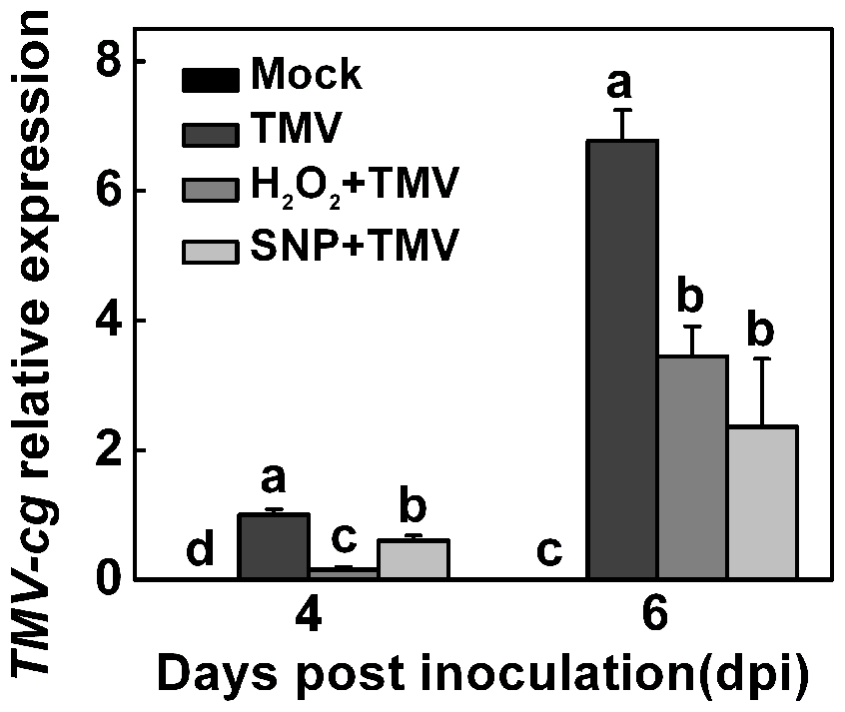
Effects of exogenous H_2_O_2_ and NO on TMV infection in *Arabidopsis* plants. Effects of exogenous application of hydrogen peroxide (H_2_O_2_) and nitric oxide (NO) and TMV inoculation on *TMV-cg* transcription in non-inoculated upper leaves of *Arabidopsis* plants. The non-inoculated upper leaves were pretreated with H_2_O_2_ or SNP 12 hours before TMV inoculation. *TMV-cg* transcript levels were analyzed by real-time reverse-transcription polymerase chain reaction at 4 and 6 days post inoculation (dpi). The results are expressed as the mean ± SD, *n*  =  4. The letters indicate significant differences between the treatments (*P*< 0.05).

Wild-type Col-0 *Arabidopsis* was also pretreated with H_2_O_2_ and SNP followed by inoculation with crucifer tobamovirus, which is related to TMV (TMV-cg, a strain of *Turnipvein*-*clearing virus*). As shown in Figure 8, in non-inoculated upper leaves, H_2_O_2_ and SNP pretreatment greatly reduced TMV-cg mRNA levels at 4 and 6 dpi compared to untreated plants that received TMV inoculation alone.

## Discussion

RDR1, a key enzyme in viral resistance, is known to be induced by viruses and SA [6], [8], [27], [28]. In the present study, we showed that *RDR1* transcript activation was accompanied by H_2_O_2_ and NO accumulation after TMV challenge in *N*. *tabacum*, *N*. *benthamiana*, and *Arabidopsis* plants. Chemical scavenging of H_2_O_2_ and NO partly blocked TMV- and SA-induced *RDR1* expression and increased TMV susceptibility. Furthermore, TMV-induced H_2_O_2_ synthesis and the associated *RDR1* expression were reduced by NO elimination and reversed by exogenous NO application, but H_2_O_2_ removal had no effect on TMV-induced NO evolution. Therefore, our results indicate that H_2_O_2_ may act downstream of NO to up-regulate *RDR1*, which contributes to the restriction of systemic infection and virus accumulation. This study provides initial insights into the signaling mechanisms underpinning virus- and SA-induced RDR1 activation in host plants.

### Involvement of H_2_O_2_ and NO in TMV- and SA-induced *RDR1* induction

H_2_O_2_ and NO are secondary molecules induced by pathogen invasion to activate defense expression and mediate SA defense responses in processes called oxidative bursting and NO bursting, respectively [18], [20], [29]. In this study, we observed a rapid and significant accumulation of H_2_O_2_ and NO in *N*. *tabacum* plants (Figure 1). Interestingly, this TMV-induced generation of NO and H_2_O_2_ was followed by *NtRDR1* transcript induction in short succession (Figure 1). These results indicate a possible connection between TMV-induced H_2_O_2_ and NO production and the induction of *RDR1* expression. Furthermore, in agreement with a previous study on *N*. *glutinosa* plants where exogenous H_2_O_2_ induced transcript expression of *NgRDR1* [13], we observed that exogenous H_2_O_2_ and NO induced *NtRDR1* transcript expression in *N*. *tabacum*, and TMV-induced *NtRDR1* expression was strongly suppressed by DMTU and cPTIO application. As the important plant signaling molecule involved in defense responses to pathogen attack, SA has also been found to elicit plant *RDR1* expression [6]–[8], and in this study, the expression of SA-induced *NtRDR1* was also compromised by H_2_O_2_ and NO scavengers. These results highlight the indispensable roles of H_2_O_2_ and NO in TMV- and SA-induced *RDR1* induction in *N*. *tabacum* plants. However, *NtRDR1* induction was not fully abolished by DMTU or cPTIO treatment (Figure 2); thus, additional unknown H_2_O_2_- and NO-independent pathways in the RDR1 signaling pathway may also exist.

The characteristics of H_2_O_2_ and NO involvement in TMV- and SA-induced *NtRDR1* expression in *N*. *tabacum* are similar in *N*. *benthamiana* and *Arabidopsis* plants (Figure 3), although the magnitude of induction/suppression by exogenous H_2_O_2_, NO, and their scavengers differed among species. In *Arabidopsis*, *AtRDR1* expression in *atrbohD* and *atnoa1* mutants was only 20% of the expression in Col-0 plants in response to TMV challenge (Figure 5C), which further emphasizes the role of H_2_O_2_ and NO in *RDR1* induction. Collectively, H_2_O_2_ and NO mediate TMV- and SA-induced *RDR1* activation in all three model host plants, and this may be a general mechanism in all plants.

### Relationship between H_2_O_2_ and NO in the *RDR1* induction signaling pathway

In *N*. *tabacum*, the NO scavenger cPTIO completely inhibited H_2_O_2_ accumulation by TMV inoculation (Figure 4). Conversely, H_2_O_2_ elimination did not affect TMV-induced NO generation. These observations suggest a possible crosstalk between H_2_O_2_ and NO, and NO may act upstream of H_2_O_2_ in modulating TMV-induced *RDR1* activation. This possibility was also supported by time-course data showing that NO generation preceded H_2_O_2_ induction in *N*. *tabacum* plants in response to TMV infection (Figure 1). This signaling pathway also seems to exist in *Arabidopsis* plants, as H_2_O_2_ accumulation was sensitive to NO, whereas NO generation was not affected by H_2_O_2_ under TMV-inoculated conditions using http://www.arabidopsis.org/servlets/TairObject?id=40002&type=locusatrbohD and *atnoa1* http://www.arabidopsis.org/servlets/TairObject?id=132871&type=locusmutants (Figure 5). Furthermore, *in planta* H_2_O_2_ concentration changes were closely related to *AtRDR1* induction, and lower *AtRDR1* transcript levels in both *atrbohD* http://www.arabidopsis.org/servlets/TairObject?id=132871&type=locusand *atnoa1* mutants were greatly increased by exogenous H_2_O_2_ treatment. In contrast, SNP pretreatment only increased *AtRDR1* transcript levels in *atnoa1* http://www.arabidopsis.org/servlets/TairObject?id=40002&type=locus plants and not in *atrbohD* plants. Therefore, TMV-induced NO likely triggers H_2_O_2_ accumulation, which plays a role in inducing plant RDR1 activation.

Exactly how NO cooperates with H_2_O_2_ to induce RDR1 remains unknown. NO inhibits CAT and ascorbate peroxidase (APX) activity to increase the level of H_2_O_2_ [30]. It has been proposed that periodic H_2_O_2_ accumulation can be attributed to the inhibition of CAT and APX by a strong early NO burst, which is accompanied by a following wave of secondary NO generation to enhance defense responses to pathogen stress [31]. H_2_O_2_ may function as a downstream amplifier to spread the signal and induce the defense response. Thus far, it is not known whether a direct interaction between H_2_O_2_ and *RDR1* occurs. Alternatively, H_2_O_2_ may activate downstream signal transduction such as the mitogen-activated protein kinase (MAPK) cascade and transcription factors to modify *RDR1* expression [32], [33]. Nevertheless, H_2_O_2_ and NO have been reported to interact in a variety of patterns, and although some studies have shown that NO treatment can induce the production of H_2_O_2_ [22], other studies have shown opposite results. For example, in cucumber plants, NO acted downstream of H_2_O_2_ in brassinosteroid-induced abiotic stress tolerance [21]. Thus, the relationship between H_2_O_2_ and NO in signal transduction may be more complicated than the simple linear manner in which H_2_O_2_ induces NO or vice versa [21]. Thus, the relationship between H_2_O_2_ and NO in signal transduction may be more complicated than a simple linear relationship in which H_2_O_2_ induces NO or vice versa [21]. In addition, H_2_O_2_ and NO may crosstalk differently under different stresses, species, and plant status. Further experiments are still needed to fully establish the relationship between H_2_O_2_ and NO and its possible function in RDR1 induction in different plants.

### H_2_O_2_- and NO-associated *RDR1* induction functions in the basal defense against TMV infection

In this study, H_2_O_2_ and NO assisted in defending against viral attack in *N*. *tabacum* and *Arabidopsis*, and DMTU and cPTIO significantly increased TMV susceptibility in *N*. *tabacum* (Figures 6 and 8). These results imply that H_2_O_2_- and NO-associated RDR1 induction may play a key role in the anti-TMV defense. Furthermore, TMV attack damaged EV plants more severely than it damaged *MtRDR1*-transformed *N*. *benthamiana* plants (Figure 7), and the natural loss of RDR1 function in *N*. *benthamiana* did not allow H_2_O_2_ and NO to diminish TMV infection in these plants, even though *NbRDR1m* was induced by exogenous H_2_O_2_ and NO. There were also no evident effects of H_2_O_2_ and NO on TMV susceptibility in transgenic *N*. *benthamiana* plants, as the *MtRDR1* gene was constitutively expressed under the 35S promoter in the transgenic plants (Figure 7). These results highlight the primary function of H_2_O_2_- and NO-associated RDR1 in the antiviral mechanism, which is contingent on functional RDR1. RDR1 was found to be involved in separate but overlapping viral resistance and post-transcriptional gene silencing mechanisms in plants [34]. A recent study using siRNA deep sequencing revealed that *rdr1-1* plants contained dramatically fewer viral siRNAs in a short time after the inoculation of TMV-cg, which clearly suggests that RDR1 contributes to the biosynthesis of TMV siRNAs [11]. AtRDR1-dependent production of CMV siRNA has also been observed when the silencing suppressor of CMV is absent [10]. In the present study, H_2_O_2_ and NO pretreatment induced a higher level of the initial *RDR1* transcript, which may have induced stronger RNA silencing and contributed to the basal antiviral defense in *N*. *tabacum* and *Arabidopsis*. Furthermore, *N*. *benthamiana* naturally loses RDR1 function to maintain a higher level of RDR6-dependent antiviral defense [34]. However, in this study, *MtRDR1*-transformed plants showed a higher constant resistance to TMV than EV *N*. *benthamiana* plants (Figure 7), and this compensation mechanism may not be sufficiently effective to compensate for the loss of RDR1.

**Figure 9:**
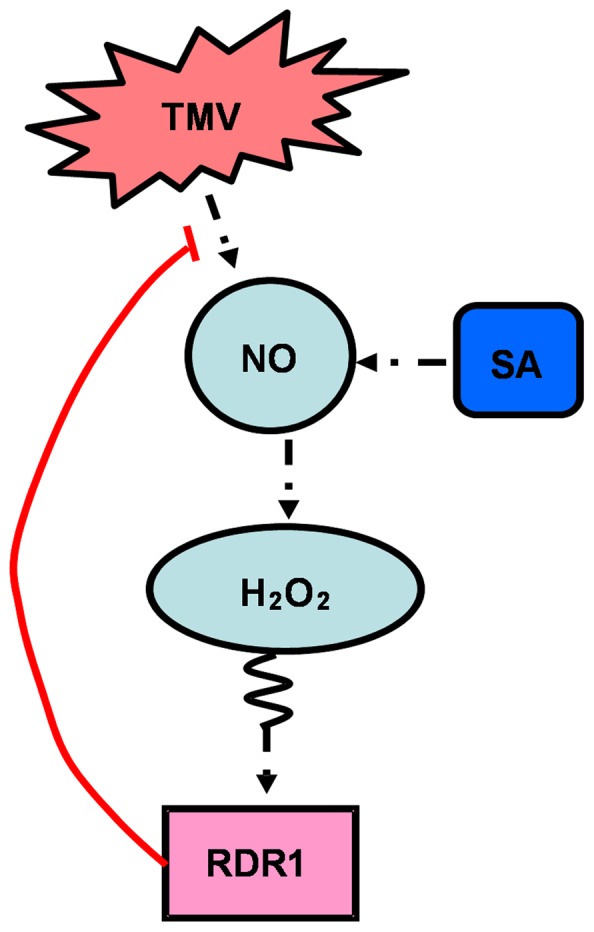
A hypothetical model for the role of H_2_O_2_ and NO in the induction of RDR1. Black dashed lines indicate positive interactions; red solid line indicates negative regulation.

In summary, we have addressed the role of H_2_O_2_, NO, and the relationship between them in TMV-induced RDR1 activation and the antiviral defense response in the model host plants *N*. *tabacum*, *N*. *benthamiana*, and *Arabidopsis*. The results strongly imply that TMV-induced NO accumulation functions upstream of H_2_O_2_ to mediate RDR1 induction, which plays a critical role in strengthening RNA silencing to restrict virus systemic infection and accumulation (Figure 9). This signaling pathway may be a general pathway common to plants.

## Materials and Methods

### Plants, viruses, and chemical treatments

Transgenic homozygous *N. benthamiana* plants with the binary vector pCAMBIA 2300 containing the *RDR1* gene from *Medicago truncatula* (R15-1) or empty vector (EV) not containing the *RDR1* transgene (V16-2) were generously provided by Richard S. Nelson (Samuel Robert Noble Foundation, Ardmore, OK). The leaf disc method was used for *Agrobacterium tumefaciens*-mediated transformation of *N. benthamiana* harboring the 35S: *MtRDR1* transgene, which was detailed in a previous study [6]. These *N*. *benthamiana* and *N*. *tabacum* plants were cultivated in a controlled growth chamber at 24°C with a 16-hour photoperiod and 50% humidity. For *Arabidopsis*, seeds of wild-type ecotype Col-0 and mutants *atrbohD* (AT5G47910) and *atnoa1* (AT3G47450) were sown on autoclaved soil and vernalized at 4°C for 3 days. Then, the plants were germinated and grown at 23 to 25°C with 16 hours of light and 8 hours of darkness. Approximately 3 to 4 weeks after germination, the *N*. *tabacum*, *N*. *benthamiana*, and *Arabidopsis* plants were chemically treated in the experiments. The plants leaves were sprayed with water or fresh solutions of 5 mM H_2_O_2_, 0.2 mM SNP, 5 mM DMTU, 0.2 mM cPTIO, 2 mM SA, 5 mM DMTU plus 2 mM SA, or 0.2 mM cPTIO plus 2 mM SA. In the experiments with a combination treatment of chemical modulator and TMV inoculation, chemical pretreatments were only applied on the upper leaves, and the lower 2–3 fully developed leaves were used for inoculation with TMV 12 hours after chemical pretreatment. Specifically, leaves of *N*. *tabacum* and *N*. *benthamiana* were inoculated with TMV (U1 strain) suspensions using cotton tips on adaxial surfaces previously dusted with carborundum powder. The viral inocula (prepared in 5 mM sodium phosphate, pH 7.5) of *N*. *tabacum* and *N*. *benthamiana* were applied at concentrations of 10 µg/mL and 1 µg/mL, respectively. The inoculation of *Arabidopsis* plants was performed using a fine sable paintbrush to apply a suspension of the TMV-cg strain at 5 µg/mL in 5 mM sodium phosphate, pH 7.5 onto leaves sprinkled with carborundum. Mock inoculations were performed with phosphate buffer only. Plants were randomly assigned to the treatments, each with four replicates. The upper, newly developed systemic leaves were collected at different time points as indicated for physiological and molecular measurements. One biological sample was obtained by pooling the leaves from three to four plants, and four biological repeats were analyzed for each treatment. The experiments were independently performed three times.

### H_2_O_2_ and NO detection and quantification

H_2_O_2_ detection using a CLSM system was conducted as described in previous studies with minor modifications [35]. Leaf sections (0.5 by 0.5 cm) were placed into a loading buffer with 50 mM Tris-KCl (pH 7.2) containing 100 µM of H_2_DCF-DA. Before further experiments were performed, the peels were preincubated in the dark for 1 hour and immediately examined under a CLSM system (Leica TCS SP5; Leica Microsystems, Wetzlar, Germany). The sections were excited using the 488-nm line of an argon laser, and dye emissions were recorded using a 505- to 530-nm band-pass filter.

H_2_O_2_ *in situ* detection was performed using a 3,3-diaminobenzidine (DAB) staining method [36]. H_2_O_2_ quantification was performed using a spectrophotometric assay at OD412 [37], [38]. The minor modifications to these experiments have been previously described [39]. The NO concentration was determined using Griess reagent (Sigma-Aldrich) [40].

### RNA extraction and transcript level estimation by real-time quantitative PCR

One biological sample was obtained by pooling the leaves from five plants, and four biological repeats were analyzed. Total RNA was extracted using TRIzol reagent (Invitrogen, Carlsbad, CA, USA) according to the manufacturer’s specifications. Genomic DNA was removed using a purifying column. Total RNA (1 µg) was reverse-transcribed using 0.5 mg of oligo(dT)_12–18_ (Invitrogen, Carlsbad, CA, USA) and 200 units of Superscript II (Invitrogen) following the manufacturer’s instruction. Gene-specific primers were designed based on the mRNA sequence for analyzing transcript levels of *RDR1* in each species. TMV-specific primers were also designed according to the sequence encoding the TMV-CP (U1 strain) and TMV genome (cg strain). The primers used are listed in Table S1.

Quantitative real time-PCR was performed using an iCycler IQTM Real-Time PCR Detection System (Bio-Rad, Hercules, CA, USA). PCR was performed using SYBR Green PCR Master Mix. PCR cycling conditions were as follows: 95°C for 3 minutes, followed by 40 cycles of denaturation at 95°C for 10 seconds, annealing at 58°C for 45 seconds, and extension at 72°C for 30 seconds. Fluorescent signals were collected during the 58°C step. To verify the amplification of a single product, a dissociation curve was generated at the end of the PCR cycles using software provided with the iCycler iQTM real-time PCR detection system. The *NtActin*, *NbActin*, and *AtActin* genes were used as internal controls, relative gene expression was calculated [41].

### Chlorophyll fluorescence imaging

Chlorophyll fluorescence was determined using PAM imaging (IMAG-MAXI, Heinz Walz, Effeltrich, Germany). Plants were placed in darkness for 30 s to measure Fv/Fm. Minimal fluorescence (F_0_) was measured during the weak measuring pulses, and maximal fluorescence (Fm) was measured by a 0.8-second pulse of light at approximately 4000 µmol/(m^2^s).

### Statistical methods

Four independent replicates were applied in each determination. The data were statistically analyzed using analysis of variance and tested for significance (*P*<0.05) using Tukey’s test.

## Supporting Information

Table S1 Primers used for real time reverse-transcription polymerase chain reaction assays. F: forward; R: reverse.
(DOCX)
