# Supplementary material for: The Role of Hydrogen Peroxide and Nitric Oxide in the Induction of Plant-Encoded RNA-Dependent RNA Polymerase 1 in the Basal Defense against Tobacco Mosaic Virus
Source: PLoS One. 2013 Sep 30;8(9):e76090. doi: 10.1371/journal.pone.0076090 (PMC3786905; doi:10.1371/journal.pone.0076090)
Supplement: Table S1 — Primers used for real time reverse-transcription polymerase chain reaction assays. F: forward; R: reverse. (DOCX) [file pone.0076090.s001.docx]

**Supporting Information**

**Table S1. Primers used for real time reverse-transcription polymerase chain reaction assays.** F: forward; R: reverse.

| **Gene** | **Encoding protein** | **Accession no.** | **Primer pairs** |
| --- | --- | --- | --- |
| *NtActin* |  | AB158612.1 | F: 5’ -CACTAGTGCTGAACGGGAAA-3’  R: 5’ -ACCTGCCCATCTGGTAACTC-3’ |
| *NbActin* |  | AY594294.1 | F: 5’ -TGCCGATAGAATGAGCAAAG-3’  R: 5’ -GCTGAGGGAAGCCAAGATAG-3’ |
| *AtActin* |  | AK230311.1 | F: 5’ -CCTCAAAGACCAGCTCTTCC-3’  R: 5’ -TCTTTGCTCATACGGTCAGC-3’ |
| *NtRDR1* | *N.tabacum* RNA-dependent RNA Polymerase | AJ011576 | F: 5’ -CAAATCAGGAATGTCGCAAA-3’  R: 5’ -GCGAACAAGCTACATCAGGA-3’ |
| *NbRDR1m* | *N.benthamiana* RNA-dependent RNA Polymerase | AY574374 | F: 5’ -TGTTGGGATCCAGACCTGGTT-3’  R: 5’-TCAACTTCCTCAATTGTGACATCAT-3’ |
| *AtRDR1* | *Arabidopsis* RNA-dependent RNA Polymerase | AY148431.1 | F: 5′-AATTGGGATGAGGAAACTGC-3′  R: 5′-ATTGCTCATCAGACCCATCA-3′ |
| *TMV-CP* | *Tobacco mosaic virus* (U1) coat protein | V01408.1 | F: 5′-TTCTTGTCATCAGCGTGGGC-3′  R: 5′-TTCGGCAGTCGTGGGGTTC-3′ |
| TMV-cg | Tobacco mosaic virus genome (cg strain) |  | F: 5′-ATGCTGTTGAAGAGCTGAATG-3′  R: 5′-GCTTGCTGGGTATGAGTGAA-3′ |
